# Preclinical Tumor Growth Delay Is More Reliable from Imaging-Based Rather than Manual Caliper Volume Measurements

**DOI:** 10.3390/biomedicines13102503

**Published:** 2025-10-14

**Authors:** Ifeanyichukwu Ogobuiro, Benjamin Spieler, Ivaylo B. Mihaylov

**Affiliations:** Department of Radiation Oncology, Leonard M. Miller School of Medicine, University of Miami, 1475 NW 12th Ave., Suite 1500, Miami, FL 33136, USA; ico8@miami.edu (I.O.); bspieler@miami.edu (B.S.)

**Keywords:** murine, growth, measurement, MRI, caliper

## Abstract

**Background/Objectives:** Tumor growth delay is frequently used in preclinical experiments evaluating oncologic interventions. While treatment response in humans is based on imaging criteria for obvious reasons, manual caliper measurement of subcutaneous tumors is standard in animal studies. In a murine tumor model treated with immunotherapy (ImT) and radiotherapy (RT), the reliability of caliper measurements was tested by comparing normalized tumor growth delay (NTGD) rates derived from caliper- and image-based volumetrics. **Methods:** A 4T1 breast syngeneic murine model was used, in which thirty animals were inoculated in the right inguinal mammary fat pad and the right axilla. One RT fraction of 8 Gy was delivered to the right inguinal tumor on day 11 post-implant, and intraperitoneal ImT (PD-1 checkpoint inhibitor) injections were administered on days 11, 12, and 14. Each animal underwent three MRI scans (days 10, 17, and 20). Caliper measurements were also performed by two independent observers on the same days. The measurements were averaged and used to estimate ellipsoid tumor volumes. The acquired MRIs were used for image segmentation and volume estimation. Tumor volumes (days 17 and 20) were normalized against the baseline pre-treatment tumor volume (day 10). NTGD rates derived from hand- and image-based volumetrics were compared to assess the reliability of caliper vs. MRI estimation. **Results:** Caliper volumes between the two observers correlated at 0.799 (Pearson, *p* < 0.001). The averaged caliper volumes correlated with MRI volumes at 0.897 (Pearson, *p* < 0.001). Absolute volume differences between caliper and MRI increased with tumor growth. NTGD-derived rates showed no correlation, with only 15% of NTGD caliper rates falling within 10% of the MRI rates. **Conclusions:** NTGD rate based on caliper volumes is a suitable measure of treatment response in preclinical studies. In the experiment described herein, caliper-derived NTGD rates did not correlate with MRI ground truth. These findings suggest that more accurate tumor volumetrics, derived from stored and verifiable medical imaging sources, should be used in preclinical assessment of oncologic interventions instead of standard caliper estimates.

## 1. Introduction

The use of murine subcutaneous tumor models for evaluating the efficacy of novel therapeutics by measuring tumor growth delay is a common approach [1]. The subcutaneous tumor model allows for relatively easy longitudinal monitoring of tumor growth delay. Calipers are commonly utilized to measure tumor volume because they are fast, inexpensive, and do not require anesthesia [2]. However, the use of calipers to longitudinally measure tumor volume has been shown to be error-prone for several reasons [3]. First, caliper measurements are commonly collected along the longest two dimensions of the tumor in the x- and y-planes, with the assumption that the z-axis is the same as the shortest dimension, creating an ellipsoidal shape, which is often inaccurate [2]. Second, measurement from the epidermis, adipose tissue, and fur (if present) introduces error and variability in tumor volume measurements [4]. These relatively simple reasons, but with far-reaching consequences, underscore the need to identify improved methods for measuring tumor volumes accurately and reproducibly in murine subcutaneous tumor models.

Several methods have emerged as alternatives to calipers for measuring subcutaneous tumor volume. Casting of the tumor region has been used to estimate tumor volume but has wide variability, and requires specific locations, such as the thoracic region, which provides a rigid base to capture the tumor volume fully in the casting [5]. Next is bioluminescence imaging (BLI), which is used to measure relative tumor volume. BLI, though relatively rapid with high throughput, only provides a two-dimensional planar format and does not capture absolute tumor volume. Also, BLI requires the tumor cell line to express luciferase, which limits its utility. Alternatively, computed tomography (CT) has been used to measure tumor volume and is more accurate than calipers [6,7]. However, CT is limited by inherently poor soft-tissue contrast when exogenous contrast materials are not used, and by its relatively high cost. Furthermore, CT imaging can introduce confounding factors during longitudinal tumor volume measurement via ionizing radiation. Lastly, ultrasound (US) imaging has been utilized. US has several advantages: it is inexpensive, noninvasive, provides good soft-tissue contrast without exogenous contrast, has high throughput with reliable tumor-volume measurement, and does not use ionizing radiation [8,9]. However, US imaging is less effective for small tumors, is susceptible to artifacts, and requires a steep learning curve for proficient use [10]. These limitations highlight the advantages of using MRI, which has been shown to capture small tumors with high accuracy and intrinsic contrast while maintaining high throughput and no ionizing radiation, making it the ground truth [11,12,13]. However, most preclinical subcutaneous tumor-volume measurements default to the use of calipers, given the high cost of MRI.

With MRI widely recognized as the ground truth for measuring tumor growth delay, the main objective of this work is to evaluate how well caliper measurements reflect MRI-based tumor-volume measurements.

## 2. Materials and Methods

The study was performed in a syngeneic, orthotopic 4T1 murine breast tumor model. The breast cancer cells were implanted in the inguinal mammary fat pad and right axilla. Subsequently, the animals were exposed to a single RT fraction of 8 Gy followed by intraperitoneal ImT injections on three consecutive days. Tumor size was then longitudinally measured by both caliper and MRI. Afterward, normalized tumor volumes were compared to assess the accuracy of caliper use.

Cell lines: 4T1, a mouse-derived breast cancer cell line, which mimics human stage IV breast cancer in BALB/c mice, was purchased from ATCC (#CRL-2539). The cells were cultured in RPMI-1640 medium (Gibco; Thermo Fisher Scientific, Inc., Waltham, MA, USA) supplemented with 10% fetal bovine serum (Gibco; Thermo Fisher Scientific, Inc., Waltham, MA, USA) and 1% penicillin–streptomycin (HyClone; GE Healthcare Life Sciences, Logan, UT, USA).

Murine model: Female BALB/c mice (*n* = 30; 8 weeks old; 18–20 g) were purchased from Jackson Laboratory (Bar Harbor, ME, USA). The animals were housed at 22 ± 5 °C on a 12 h light/dark cycle and fed rodent chow and water ad libitum at the University of Miami research animal facility. All experiments were approved by the Institutional Animal Care and Use Committee (IACUC), protocol 17-214-ad02 EDR.

**Figure 1:**
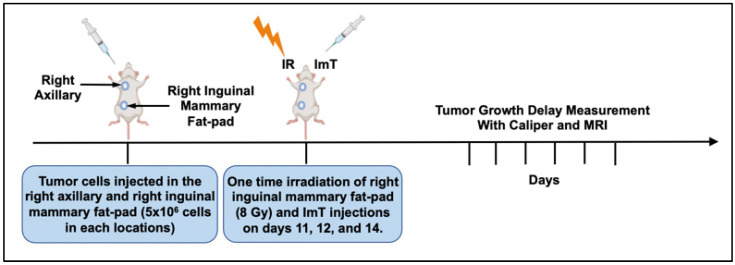
Experimental design of in-vivo experiments aimed to evaluated accuracy of tumor growth delay volume measurement of caliper compared to MRI after irradiation and immunotherapy.

Experimental design: The experimental timeline is presented in Figure 1. First, 5 × 10^6^ 4T1 breast cancer cells in a 100 μL mixture were injected orthotopically into the fifth mammary (primary tumor) pad on the right side and subcutaneously on the upper shoulder (abscopal tumor) on the right side of BALB/c mice. A single 8 Gy fraction of radiation therapy was delivered to the right inguinal mammary fat-pad tumor on day 11 post-implant, and intraperitoneal injections of anti-PD-1/CTLA-4 at 20 mg/kg were administered on days 11, 12, and 14. Each animal underwent caliper- and MRI-based tumor-volume measurements on days 10, 17, and 20. Tumor volumes measured on days 17 and 20 were normalized to the baseline pretreatment tumor volume (day 10). Normalized tumor growth delay (NTGD) rates derived from caliper- and MRI-based volumetrics were compared to assess the reliability of calipers versus MRI, as previously shown [14,15]. Of note, NTGD is defined as the fractional change from baseline: NTGD = V_t_/V_0_, where V_0_ = tumor volume at baseline (day 10) and V_t_ = tumor volume at follow-up (day 17 or 20). Thus, NTGD = 1 means no change; NTGD > 1 indicates growth; and NTGD < 1 indicates shrinkage.

Caliper measurements of subcutaneous tumors: A digital caliper was used to measure each subcutaneous tumor while the mice were conscious. The two longest perpendicular axes in the x- and y-planes of each tumor were measured by two independent observers. The y-axis was assumed to be the same as depth, and tumor volume was calculated using Equation (1), a standard practice.

V_st_ = xy^2^/2 (1)

MRI imaging and data analysis: Animals were subjected to magnetic resonance imaging (MRI) on days 10, 17, and 20 after tumor inoculation. Mice were kept under anesthesia using ketamine administered intraperitoneally at 90 mg/kg, pre-mixed with xylazine (#59399-110-20; AnaSed Injection, Akorn, Lake Forest, IL, USA) at 4.5 mg/kg. Throughout imaging, body temperature was maintained using HotSnapz reusable heating pads. At the end of the procedure, atipamezole hydrochloride (Zoetis) was injected subcutaneously at 7.5 mg/kg to aid recovery from anesthesia. After injection, the animals were placed back in their cages for recovery. For MR imaging, a T1 sequence with a voxel size of 0.5 × 0.5 × 0.5 mm^3^ was used on a 3T Siemens TrioTim scanner (Siemens Medical Solutions, Malvern, PA, USA).

Statistical analysis: Non-normalized and normalized caliper- and MRI-based tumor-volume measurements were compared, and differences were considered statistically significant at *p* < 0.05. Pairwise linear fits were plotted, with Pearson *R*^2^ computed in Excel. Statistical analyses were performed in Prism version 9.1.2 (GraphPad Software Inc., San Diego, CA, USA).

## 3. Results

**Figure 2:**
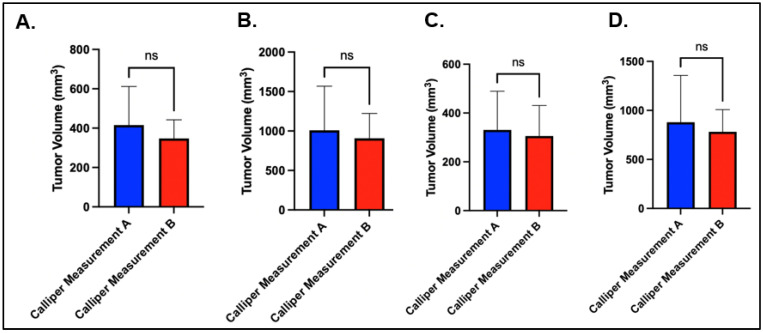
Caliper-based tumor measurements comparing technicians A and B for: (**A**) day 10 (treatment onset) right axillary; (**B**) day 20 right axillary; (**C**) day 10 right mammary fat pad; (**D**) day 20 right mammary fat pad.

The technician variability was relatively small for both the axillary and the mammary fat-pad tumor volumes. For the right axillary site, the average caliper measurement on day 10 for technician A and technician B was 415.93 mm^3^ (284.9–487.70 mm^3^) and 347.54 mm^3^ (269.50–400.5 mm^3^), respectively (*p* = 0.08), and the average caliper measurement at day 2 was 1008.88 mm^3^ (486–1206.83 mm^3^) and 906.75 mm^3^ (630.2–1177.68 mm^3^), respectively (*p* = 0.50) (Figure 2A,B). For the right mammary fat pad, the average caliper measurement at day 10 for technician A and technician B was 330.75 mm^3^ (203.15–425.30 mm^3^) and 305.77 mm^3^ (210.8–401.45 mm^3^), respectively (*p* = 0.48), and the average caliper measurement at day 20 was 879.92 mm^3^ (497.13–1044 mm^3^) and 781.47 mm^3^ (599.75–928.23 mm^3^), respectively (*p* = 0.43) (Figure 2C,D).

**Figure 3:**
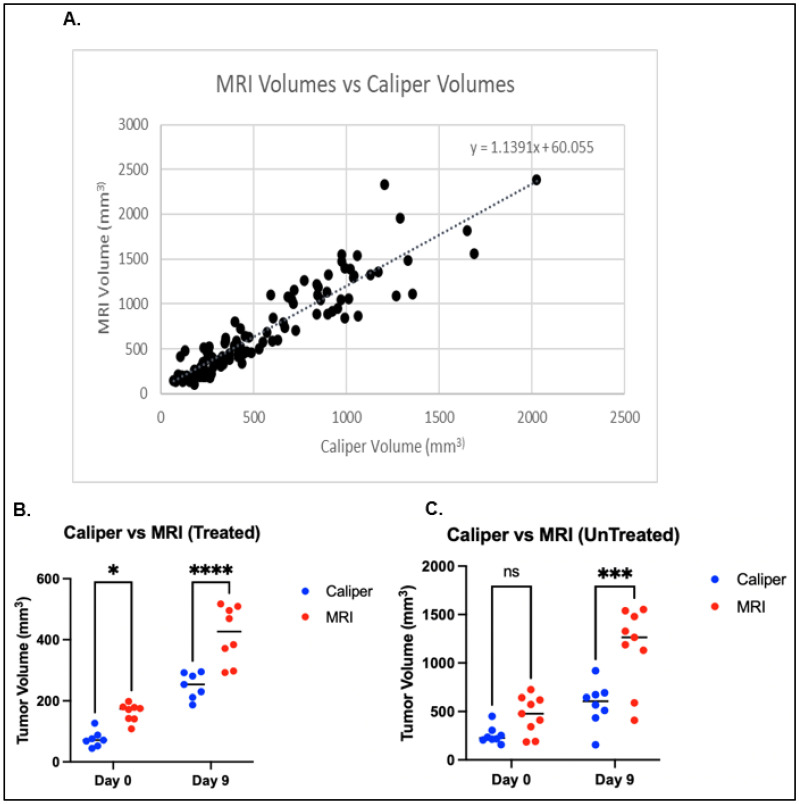
MRI volumes versus caliper-based volumes. (**A**) Tumor volume measured by MRI versus caliper. (**B**) Tumor volume measured by MRI versus caliper in treated (radiation and immunotherapy) tumors. (**C**) Tumor volume measured by MRI versus caliper in untreated (no radiation or immunotherapy) groups. ns means no statistical significance, while asterisk indicates statistical significance.

Correlation and Bland–Altman analyses were performed to compare tumor growth-delay volume calculated from caliper measurements (using the ellipsoidal formula) versus estimation by MRI. Analysis of 30 paired caliper and MRI measurements, with interquartile ranges of 183.78–664.35 mm^3^ and 274.18–842.09 mm^3^, respectively, yielded a correlation coefficient of 0.897 (Pearson, *p* < 0.001) (Figure 3A).

Although there was a positive correlation in tumor volume, there were significant differences in the absolute tumor sizes between caliper and MRI. On day 10, the mean tumor volumes measured with calipers and MRI were 254.78 mm^3^ and 462.93 mm^3^, respectively. On day 20, the mean tumor volumes measured with calipers and MRI were 574.49 mm^3^ and 1164.29 mm^3^, respectively.

**Figure 4:**
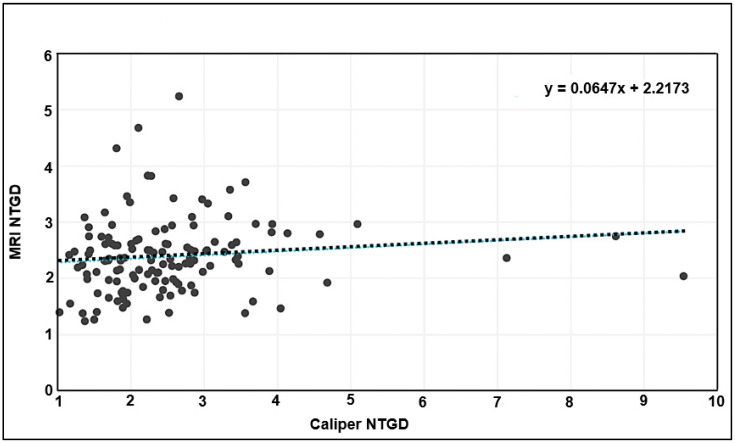
MRI versus caliper-based NTGD (pairwise). Each point represents a single tumor from the same mouse plotted pairwise—x-axis: that tumor’s caliper NTGD; y-axis: the MRI NTGD for the same tumor. (Each mouse has two tumors—right axilla and right inguinal mammary fat pad.) The dashed line shows the linear fit (y = 0.0647x + 2.2173), with Pearson *R*^2^ = 0.014.

Tumor volumes derived from caliper and MRI measurements were then normalized to the initial tumor volume from the day before treatment was initiated. When the normalized caliper- and MRI-based measurements were tested for correlation (as in Figure 3 for absolute volumes), no correlation was found (Figure 4).

## 4. Discussion

Preclinical experiments evaluating oncologic interventions rely on longitudinal tumor-volume measurement. Although MRI is widely considered the ground truth for tumor-volume assessment, calipers are commonly used for subcutaneous tumors because they are inexpensive, fast, and easy to apply. The unanswered question is whether caliper measurements faithfully reflect MRI-based tumor volume. In this study, we demonstrate that caliper-derived tumor-volume measurements do not reflect MRI-based measurements using both normalized and non-normalized growth-delay analyses.

As expected, caliper measurements show a positive correlation with MRI tumor-volume measurements. Interestingly, as shown in Figure 3B,C, there is a more dramatic difference in tumor size, with MRI capturing a much larger volume than calipers. This is concerning, given that longitudinal caliper measurements increasingly depart from the ground truth over time. Additionally, when tumor volume is normalized, the positive correlation is lost (Figure 4), and a large fraction of caliper measurements show significantly higher size differences compared with MRI.

The discordance between caliper and MRI tumor-volume measurements demonstrates inaccuracy in the use of calipers. This warrants a closer look at whether the low cost of calipers justifies the loss in accuracy. This discrepancy could be a major contributor to the challenges of translating preclinical studies to clinical trials. Several studies fail in clinical trials, even when therapeutic benefit is demonstrated in mouse models [16]. Understandably, multiple factors contribute to this disconnect; however, there is a need to focus on addressable factors. Lack of reproducibility in monitoring tumor-growth delay in preclinical models has been identified as a significant factor [17]. Therefore, standardization of MRI-based tumor-volume measurement in preclinical research should be considered. Alternatively, caliper measurements should be supplemented with histopathologic assessment at the time of sacrifice. Although this provides only a single time point, it is effectively the gold standard for response evaluation and is warranted, given the variability and uncertainty of caliper-derived volumes and subsequent response estimates.

This study has a few limitations. Although technicians A and B’s caliper measurements were not significantly different—suggesting consistent skill—we do not know whether their technique represents the broader population of personnel proficient in caliper tumor-volume measurement. Thus, the significant differences observed between caliper and MRI could reflect suboptimal proficiency with calipers. This could be addressed by creating a standardized training and assessment protocol across research institutions and then repeating the MRI–caliper comparison only with technicians who pass the proficiency assessment. Also, this study did not address the cost–benefit analysis of MRI versus calipers. Undoubtedly, MRI is more expensive, but it is more precise, more reliable, and, importantly, provides a permanent record that can be re-examined in the future, if needed.

## 5. Conclusions

This study shows the inaccuracy of caliper-based measurements compared with MRI. Addressing this issue has the potential to improve the translatability of preclinical research and increase the efficiency of moving discoveries from bench to bedside.

## Data Availability

Data can be obtained from the corresponding author.

## Abbreviations

The following abbreviations are used in this manuscript:

ImT	Immunotherapy
RT	Radiotherapy
NTGD	Normalized tumor-growth delay
BLI	Bioluminescence imaging
CT	Computed tomography
US	Ultrasound
MRI	Magnetic resonance imaging
